# Reviewers do matter

**DOI:** 10.1186/s40902-018-0140-x

**Published:** 2018-01-25

**Authors:** Seong-Gon Kim

**Affiliations:** Department of Oral and Maxillofacial Surgery, College of Dentistry, Gangneung-Wonju National University, 7 Jukhyun-gil, Gangneung, 210-702 South Korea

The publication of robust scientific articles is the most important mission for editors. Editors should bear in mind that their selections can contribute to the progress of science and their selections should be shared with all relevant parties. We cannot deny that skepticism is a step toward advancement. To resist established concepts, a pioneer may face much resistance. New findings should be proven by experiments and debates that can be performed on the basis of evidence. Reviewers are selected from a specific field. As those in the reviewers’ pool are respected in their fields, their reviews are generally logical and objective. Thus, their reviews have been of great assistance for the editors’ decisions. Thus, why do reviewers matter?

Recently, ethical publication has been stressed because of scientific scandals. Duplicated publications and the unauthorized inclusion of authors’ lists are further examples of misconduct [1]. These articles were not screened by reviewers in the 1990s. Due to recent progress in tools, duplicated publications can be screened using software. Many publishers request the email addresses of all authors to prevent unauthorized inclusion in the authors’ lists. In Korea, Hwang’s case was notorious, and these incidents are related to the manipulation of data. This type of misconduct has been poorly screened by reviewers. What happens if reviewers are compromised?

As far as it can be surmised, there is no official process for screening reviewers’ misdeeds, and there has been no classification of reviewers’ misconduct. What kinds of reviewers’ mistakes are possible? Historically, many important scientific articles have been rejected for publication because of reviewers’ opinions [2]. Most of the reviewers had intentionally ignored important evidence in the articles. To impede competitors’ publications, reviewers may demand major revisions or rejection by the editor. To prevent this type of misconduct, authors may provide information about reviewers who should not review their submissions. However, authors may not know all the potential competitors in their fields. For these reasons, some journals such as *Scientific Reports* evaluate the novelty of submissions in the publisher’s independent department. The journal’s reviewers are asked to evaluate only scientific soundness. This is important progress for the peer review process.

Most journals adopt the peer review process and require multiple reviewers for the final decision to publish. If two reviewers recommend an article for publication and one reviewer rejects it, what kind of decision is possible by the editor? What happens if the opposite situation occurs? Can the scientific facts be proven by democratic vote? This depends on the journal’s policy. Most journals require the agreement of the reviewers for the acceptance of an article. If one reviewer has a different opinion, the article may enter an eternal cycle of reviews. In such cases, the authors may seek other journals to avoid any delay in their publication. This is stressful and a waste of time for the authors.

At this point, the editor’s role becomes important. The editor should not decide the fate of an article in accordance with the reviewers’ opinions. The publisher, Springer, provides some guidelines for poor reviews. If the review process is too immediate after an article is received, the reviewer may not read the article carefully. Illogical reviews may be caused by the improper selection of reviewers. The current level of science is highly segmented. Accordingly, the gross classification of science makes it difficult to select proper reviewers. Some reviewers request the inclusion of their own articles as references to improve their own citation records [3]. Although they were selected as reviewers because of their specialty, the forced inclusion of their own articles was not deemed to be ethical. If the reviewer added new critics in each round of review, the author may be required to perform endless corrections. If the reviewer did a careful review, all of the critics should be shown the first round of review. Adding new critics in the second round of review means that the reviewer’s initial review was careless.

*Maxillofacial Plastic and Reconstructive Surgery* respects every submission, and we are concerned about irrational reviews. For a fair review process, all reviews are carefully screened by the editors. Even if a reviewer rejects an article, it may not ultimately be rejected. If the level of the review does not meet our journal’s criteria, the editor may send the article to another reviewer. Although the majority opinion on an article is generally respected, minority reports may also be considered. The editor has the right and the responsibility for the final decision. Accordingly, the editor is mainly responsible for each publication.
